# Personal Recollections of a Dentist of the Early Days

**Published:** 1884-12

**Authors:** L. W. Bristol

**Affiliations:** Lockport, N. Y.


### PERSONAL RECOLLECTIONS OF A DENTIST OF THE EARLY DAYS.

BY DR. L. W. BRISTOL, LOCKPORT, N. Y.

READ BEFORE A UNION MEETING OF THE SEVENTH AND EIGHTH DISTRICT DENTAL SOCIETIES OF THE STATE OF NEW YORK, HELD IN ROCHESTER, OCTOBER 28 and 29, 1884.

Published at the Request of the Societies.

The practitioners and students of dentistry of the present day know little of the difficulties and disadvantages under which those practicing fifty years ago labored. There were no dental colleges, no dental depots, no dental instrument makers, and in the country no regularly settled dentists. They traveled about with their "kit" under their arm.

The teeth used in those days were human teeth, procured from the battlefields or hospitals, preserved in jars with spirits of camphor. They used to say of a prosperous dentist: "Why, he is rich; he has a whole keg of teeth." They also used the teeth of neat cattle, or carved them out of a block of hippopotamus ivory, leaving the enamel in front, like the sample I will show you.

Dental laboratories were sealed sanctums, within which no other dentist was ever admitted. Dentists never recognized a lady or a clergyman in public, no matter how intimate they were in private, for people would say "he has been putting in teeth for them."

There were fanatical people in those days, just as there are now, only more so. They quoted the passage of "An eye for an eye, and a tooth for a tooth." The Quakers, my ancestors, had these words always upon their lips, arguing that if it had pleased Divine Providence to remove a tooth, or an eye, or the hair, it was sinful to oppose the will of God by an attempt to replace it by artificial means.

If a man in those days desired to enter the dental profession, and thought he had ingenuity and mechanical skill enough, all he had to do was to throw down the lap-stone, hang up the strop, throw away the press-board and goose, or the printer's rule, and go in on his nerve. In the first place he had to make his instruments, and this required mechanical skill. If he found a piece of work that some other dentist had done, you may be sure he examined it carefully and closely. I well remember that in 1833 I went three miles to see a man who had two false teeth. They were made of "seahorse," and attached to adjoining teeth with fine gold wire that had cut the teeth they were attached to nearly one-third off.

I have instruments in my possession to-day that I made forty years ago, and they are yet doing good service. Many an engagement have I filled by sneaking around just at daylight, by the back way into a back chamber, to prepare and insert two or three teeth for a young lady, making a long, tedious day of it, always stipulating that the cross dog should be chained or shut up.

We first, by measurement, fitted a block of the right curve to fill the space to be supplied with teeth, and then, with a camel's hair pencil dipped in rouge mixed with alcohol, painted the gum and pressed the block on to receive the red impression, then carved, scraped, gouged, and dug, painted and tried again, and so on, until a perfect fit was secured. We then proceeded to carve out the teeth, leaving the enamel in front. It was rude looking, but it filled the bill. We saved every root, and pivoted to it, sometimes fitting six teeth to two roots. If the roots were gone, we tied the blocks in with silk thread, or gold wire.

I have seen a block of six teeth pivoted to the canine teeth, worn until they could be removed and cleaned, roots and all, then put back and worn for a long time afterward without any apparent inconvenience or inflammation. You see that the new process of bridging in blocks of teeth is no new theory. I well remember when a great discovery was made; that of taking an impression of the mouth with wax, and pouring in calcined plaster of Paris. Oh! that was a most wonderful discovery, and it was kept a secret for a long time by the knowing ones; but it finally leaked out. If we happened to meet another dentist, and he was anyways inclined to be social, you may be sure we "set 'em up" frequently, and when he was "full" we wormed the secret out of him, if he had one.

Following the carved work came the old French Bellah teeth, of which I will show you specimens. They were mounted on gold plate with a dowel pin soldered to the plate, and this soldered to the platina clamps baked in the tooth. You will see that they were opaque; a mudy hue; no life-like shade. Still they did not look bad in the mouth of an aged person. Then came the Stockton pivot teeth. They were a great improvement in their life-like appearance. Dentists advertised the "Silicious incorruptible teeth," "diamond," "pearl," and lots of other fancy names. We struck up a plate, soldered the gold pins, and attached the teeth with hickory plugs; immersed them in water twelve hours or more, and then with a good deal of anxiety removed the plate and examined to see how many had burst by the swelling of the wood. We always directed our patients to keep them wet. If the patient was ill, and by carelessness the teeth were suffered to get dry and tumble off, they were brought back to be again put on where they belonged, and it was quite a job sometimes to get them right.

Stockton sent out his teeth by the peck. I remember that an old man used to come around about once in six months, with an old striped bag full of them. He would pour out about a couple of quarts, and we would make our selection.

Next came the single gum teeth of Stockton, with platina pins baked in the tooth. When Stockton first manufactured his single plain and single gum teeth, with platina pins, to be backed with gold and soldered to the plate, he kept it a secret. He had a large stock of pivot teeth on hand. He sent out peddlers in every direction, put the price down to ten cents each, for Mr. Stockton was "going to change his business." The agents did not say directly that he was going out of tooth making, but hinted as much. The consequence was that every dentist laid in a big stock, used all his ready money, and borrowed more to buy teeth.

In about one month other agents came around with the improved teeth. We were all sold; had to abandon the old pivot teeth and use the new. Then there was some tall swearing at the trick. Tieman, the instrument maker of Chatham Street, had bought a heavy stock. You ought to have heard that old descendant of father Abraham curse Stockton. But it was of no use. He had to succumb to the inevitable.

About 1834 the dentists began to establish offices, or rather rooms, usually at their residences or boarding houses. Ladies and gentlemen, and sometimes a clergyman, were seen calling on them. If a student desired to learn the profession he had to pay liberally for his instruction. I paid a dentist, whose office was under the printing office in which I was employed, fifty dollars for the privilege of getting into his office for one year. He was to answer any question pertaining to the business—truthfully if he could—and I was to come whenever he called me to hold his patient's head when a tooth was to be extracted. Dentists in those days never attempted to extract teeth without some one to hold the head.

It has been said that history repeats itself. In those days we used to replant and transplant teeth; but we soon found we not only transplanted teeth, but scrofula and other diseases, and the practice was declared "malpractice" and abandoned. In filling teeth we used lead, pounded very thin, and tin foil. As for gold, the only wonder is that we made as permanent fillings as we did; the foil was so harsh and brittle. We always filled the teeth so as not to have the fillings show. If they did, or a gold clasp was visible, our patients always found fault. How changed the tastes of people now. Richmond gold crowns and contour fillings would then have been declared unnatural. If a tooth was gone we tried to replace the same with one as near the shape and color of the other teeth as possible.

About the year 1833 or 1834, I have lost the exact date, the notorious Crawcours of London and Paris opened an office in New York, and for six or eight months made a small fortune by filling teeth with amalgam, asserting its qualities to be that of "making a stump into a sound tooth at once, as if by magic!" They called the compound "the Royal Mineral Succedaneum." These Crawcours were the father, mother, brother, and sister of the amalgam practice. They used the old French five franc pieces, filed up and mixed into a soft paste with mercury. Many people who had good, serviceable gold fillings, that had stood a long time, had the gold dug out and the amalgam substituted, "because the gold showed." These Crawcours had a successful run of it. They were not dentists, and knew but little of the profession. When they found that warrants were out for their arrest they dug out, took a packet ship, and were well out past Sandy Hook before the officers got after them. They brought amalgam into bad odor, but after a few years the profession got to using it again, although there then was, and is now, a good deal of opposition to it. It is only within a very few years that we have learned to properly use it, and to succeed in making good fillings.

Of the bases for artificial dentures we have had gold, silver, porcelain, gutta-percha, pyroxylene, rose pearl, aluminum, platina, celluloid, rubber, and Josiah Bacon. In 1838, when I was located in Syracuse, practicing with Dr. Bliss, there came into the office one afternoon a lady who showed us a set of gum block teeth made by Dr. Thos. Harrison, of Lockport. They looked natural, fitted well, and it was the best piece of work we had ever seen of the block gum teeth. Bliss said to me: "You are young and have no family; suppose you go to Lockport, get acquainted with him, learn his method, and come back, and we will form a partnership and do that kind of work." I thought the matter over and decided to do so; I packed my gripsack, took the grand old Erie Canal for Lockport, where I arrived in good time, and found Harrison was at Lewiston. I took the stage for that place, where I arrived about eleven P. M., and found him at the Lewiston hotel, lying in the corner of the bar-room, behind the stove, pretty drunk. The prospect did not look promising, by any means. Next day I made his acquaintance, told him what I was after, and proposed to help him. He consented, and I assisted him to sober up, got his hair cut, bought him a new suit of clothes, and he made quite a respectable appearance. We went to Lockport, took a back room in the third story of a block, put up a furnace and commenced business. But I found that the work did not stand the test of time in the mouth. The pieces were porous, absorbed the saliva, and soon became discolored. We could not repair by reheating, for they would fly all to pieces, and pop like corn. He kept no record, never weighed the ingredients, guessed at the amount, and as a consequence never had two bodies alike.

Here let me say that I have spent more time, passed more sleepless nights sweating over a hot furnace, trying to surmount difficulties, or making block gum teeth moulded over a cast of the mouth, and to be worn in the same mouth from which the cast was taken, than over any other branch of the profession, without ever having succeeded or been thoroughly rewarded, and I never expect to be; the shrinkage, warping, checking, bulging, never has been overcome. The composition of the block must be so equalized that while some of the materials shrink others must oxydize and increase in bulk. This retains the shape of the cast, but makes the body porous. Then you rely on enameling to fill the interstices. It will do this, in a measure, on the surface, but not all through the body, which will absorb the fluids of the mouth, with some patients more than others.

John Allen, of New York, came the nearest to perfection of any man, but his work failed in time. His method had the advantage of using teeth already baked. A platina plate was swaged, the teeth backed with platina and soldered to the plate with fine gold foil. The interstices were filled with a granulated body, the picee invested in asbestos and plaster paris, and baked. Then it was enameled and baked again; but to an experienced eye, in a strong light, cracks or checks would be apt to show on cooling. By oiling the block the checks would not show; but time and human saliva are very searching, and when a plate began to give out it went all to pieces. It had one advantage; it could be re-enameled and baked again.

About 1835 or 1836 Creosote was introduced to the public, and created a good deal of interest in the medical and dental profession. Some dentists went so far as to say that they had abandoned extracting teeth, and one in Syracuse advertised in large letters on the awning post, the whole length of a block: "Toothache cured by Creosote without extracting." He charged one dollar for introducing a small pledget of cotton saturated with Creosote, and covered with gum mastic and turpentine.

A great many discoveries and some valuable improvements were made. One dentist, in an eastern city, had very delicate instruments with which he "cut the ligamentum dentatum," and the teeth tumbled out easily, and our patients would ask us, "have you got an instrument to cut the ligamentum dentatum?"

In 1837, a dentist by the name of Jacks, of Catskill, invented and patented a double air-chamber plate along the alveolar ridge. This chamber was perforated with one-eighth inch holes about half an inch apart, and soldered to the main plate. The patient sucked the gum into the holes as one would suck a key on the lips, and, if worn long enough, the "teat" would become strangulated and drop off, remaining in the air chamber, which would also become filled with food, mucous, etc. There was no way to get at it to cleanse it, and it made one of the most filthy dental structures imaginable to mend, and forced one to open his doors and windows to let the stench out.

Another dentist patented flexible rubber air chambers, and sold the exclusive right to any dentist in the locality who would pay the highest price. The flexible had quite a run for a time, but the rubber decomposed in the mouth, and the dentist soon had all he could do repairing plates broken through the center, and the patent went into oblivion.

Some one has said that instead of going to California to-day for gold, it would be better to go to our cemeteries and get the gold buried with the dead. I have taken some pains to examine the assertion, so far as my own practice is concerned, and I have found that from 1839 to the introduction of rubber work, the average amount of gold plates, gold solder, and gold foil used in my practice amounted to a trifle over thirty-one dollars each month. One can see that if others in the profession used the same amount (and many, probably, used more), a vast amount of gold is buried in our cemeteries.

In 1848, Morris Levett patented an enamel for coating a silver or gold plate the color of the natural gums, doing away with gum teeth. A man by the name of Englebright was the agent to sell the same. He was a sweet talker, and showed many specimens of gold and silver plates enameled. They looked well, and seemed to be a desirable improvement. Levett said he had sold over forty thousand dollars' worth in the last three months. He came to Buffalo, visited the dental offices, and offered to sell office rights, but preferred to sell territories. We met at the office of a prominent dentist to see the enameling process. I was requested to conduct the experiment. While doing so, first one would retire with the vendor, then another; finally the dentist in whose office we were retired and had a private talk, and when they returned we were told that the doctor had purchased the exclusive right to the county of Erie, and if we wanted an office right we must apply to him. He had not made up his mind yet what would be the price, but would let us know in a few days. We all looked chap-fallen, took our hats and stepped down and out. After I retired to bed, the thought struck me that the enamel I had used in making artificial eyes might answer the purpose. The next day I tried it, and found it worked equally as well as Englebright's. I enameled a plate, showed it to him, and he would not believe but that I had used Levett's, until I enameled another piece in his presence, when he was satisfied that Bristol's artificial eye enamel would do as well as his.

He had taken in part payment for the exclusive Erie County right a gold watch valued at one hundred dollars. He gave half of an office right to me for Lockport, valued at twenty-five dollars, if I would keep still. Having occasion to visit Lockport, where my family resided, I found Englebright there. He had got the dentists all excited. I had given him an order on my partner for his half of an office right. In the evening they all met at my old office, and again I was requested to conduct the process. While doing so the old game was repeated; first one would retire with the agent, and then another. Finally Dr. O. W. May offered him his house and lot for one-half of the State of Pennsylvania. I tried to stop May, but it was of no use. He was bound to get rich out of the enamel. Englebright called me out and said: "Your family is sick, and you know too much. Take this gold watch and clear out. Stay away from Lockport one week." I pocketed the watch and left, next day returning to Buffalo. In about a week Englebright returned to Buffalo. In the meantime one Buffalo dentist had written east, and found the thing a humbug, and threatened the agent with prosecution for fraud. They finally compromised by the agent giving back the watch and part of the money, he taking back the right to Erie County. He came to me and said if I would give up the watch he would have the right assigned to me. I did so, and am still the sole owner of the right to Morris Levett's enamel for Erie County. If any dentist in Buffalo wants an office right, let him come to the captain's office, and I will give him a license to enamel for life, free of charge. Don't you call that liberal?

When I look back along the pathway I have trod in dentistry, it is gratifying to observe the great improvements that have been made from time to time. The profession, from a small number of self-educated men, jealous of each other, has now become a multitude of liberally educated men, ready to impart to one another all desirable discoveries and improvements, all trying to elevate the practice. It is especially gratifying to me that I was one of the four men who shook hands on the platform of the depot at Trenton Falls, pledging ourselves to go home and set to work to found "the Western New York Dental Association," and to invite all practicing dentists west of Cayuga Bridge. This we did, and the call was signed by B. T. Whitney, Buffalo; Geo. E. Hays, Buffalo; E. F. Wilson, Rochester; L. W. Bristol, Lockport.

At the first meeting B. T. Whitney was elected president. We had a largely attended meeting, and a kind of dental love feast, at which some acknowledged that they had done things in the past that were unprofessional, but they would do so no more.

That association was the entering wedge that culminated in our present State society and Law. Two of the men that signed that call have gone to their long home, honored and beloved by all their professional brethren, and their memory will always be cherished. Two are left to hope and pray that a still brighter and better future is in store for those practicing the profession throughout the United States.

I have had my day, and it has, in a measure, been a stirring one. I am now getting old, and my locks are whitened. You have urged me, before I shall have left you forever, to give you some of the reminiscences of early dentistry. This paper is in response to that request; it is necessarily a thing of shreds and patches, but it will give you some idea of what we older ones have passed through, scenes never to be re-enacted. We old heads must now step down and let you younger men have your day, and I hope it will be a better one than ours has been.

